# Relationship between liver fat content and lifestyle factors in adults with metabolic syndrome

**DOI:** 10.1038/s41598-022-22361-3

**Published:** 2022-10-19

**Authors:** Saara Laine, Tanja Sjöros, Taru Garthwaite, Maria Saarenhovi, Petri Kallio, Eliisa Löyttyniemi, Henri Vähä-Ypyä, Harri Sievänen, Tommi Vasankari, Kirsi Laitinen, Noora Houttu, Ekaterina Saukko, Juhani Knuuti, Virva Saunavaara, Ilkka H. A. Heinonen

**Affiliations:** 1 Turku PET Centre, University of Turku, Åbo Akademi and Turku University Hospital, Turku, Finland; 2 Department of Clinical Physiology and Nuclear Medicine, University of Turku and Turku University Hospital, Turku, Finland; 3 Department of Biostatistics, University of Turku, Turku, Finland; 4 The UKK Institute for Health Promotion Research, Tampere, Finland; 5 Institute of Biomedicine, University of Turku, Turku, Finland; 6 Paavo Nurmi Center, University of Turku, Turku, Finland; 7 Division of Medical Imaging, Department of Medical Physics, Turku University Hospital, Turku, Finland; 8 Faculty of Medicine and Health Technology, Tampere University, Tampere, Finland; 9 Department of Radiology, Turku University Hospital, Turku, Finland; 10 Rydberg Laboratory of Applied Sciences, University of Halmstad, Halmstad, Sweden

**Keywords:** Endocrine system and metabolic diseases, Hepatology, Magnetic resonance imaging

## Abstract

The aim of this study was to investigate the associations between liver fat content (LFC), sedentary behaviour (SB), physical activity (PA), fitness, diet, body composition, and cardiometabolic risk factors in adults with metabolic syndrome. A total of 44 sedentary adults (mean age 58 [SD 7] years; 25 women) with overweight or obesity participated. LFC was assessed with magnetic resonance spectroscopy and imaging, SB and PA with hip-worn accelerometers (26 [SD 3] days), fitness by maximal bicycle ergometry, body composition by air displacement plethysmography and nutrient intake by 4-day food diaries. LFC was not independently associated with SB, PA or fitness. Adjusted for sex and age, LFC was associated with body fat%, body mass index, waist circumference, triglycerides, alanine aminotransferase, and with insulin resistance markers. There was and inverse association between LFC and daily protein intake, which persisted after further adjusment with body fat%. LFC is positively associated with body adiposity and cardiometabolic risk factors, and inversely with daily protein intake. SB, habitual PA or fitness are not independent modulators of LFC. However, as PA is an essential component of healthy lifestyle, it may contribute to liver health indirectly through its effects on body composition in adults with metabolic syndrome.

## Introduction

Obesity is associated with an increased risk of developing non-alcoholic fatty liver disease (NAFLD)^1^. In NAFLD excess triglycerides accumulate in the hepatocytes increasing liver fat content (LFC), which may cause inflammation and damage the liver^1^. NAFLD is associated with metabolic syndrome (MetS) and is described as the hepatic component of this condition^2^. MetS is a complex disorder characterized by elevated fasting plasma glucose and triglycerides levels, hypertension, low HDL levels, and a large waist circumference [WC]^3^, and it increases the risk of cardiovascular diseases and type 2 diabetes^4^. The current obesity pandemic is estimated to lead to 25% of the global population eventually developing NAFLD, which is identified as one of the growing causes of liver cancer^5^. NAFLD is usually asymptomatic and becomes apparent when the situation is already severe^6^. Therefore, early diagnosis and prevention play important roles in the detection and treatment of this disease.

Sedentary behaviour (SB) is associated with unhealthy body composition^7–9^, whereas habitual PA is associated with lowered body fat%, body mass index [BMI] and WC^10,11^. Previous studies have shown that LFC is positively associated with SB and inversely with PA, and this association is strengthened in a dose-dependent manner^12–17^. However, these studies were based on self-reports, which can overestimate actual SB and PA levels^18^. Further, in a majority of these studies LFC was determined by ultrasound or fatty liver index, instead of the gold standard LFC assessment methods liver biopsy and magnetic resonance spectroscopy (MRS). To our knowledge, only few studies to date have investigated the associations between accelerometer-measured SB and PA and liver health^19–21^. The duration of accelerometry measurement has generally been only 4–7 days, however, which might not accurately represent habitual activity and behavior over longer periods of time^22^. Additionally, cardiorespiratory fitness and diet were assessed only in one study^19^.

Therefore, the primary aim of this study was to comprehensively and simultaneously investigate important lifestyle factors and liver health, measured as LFC. We particularly wanted to examine the associations between LFC and SB and habitual PA in sedentary adults with MetS, who are thus at increased risk of developing metabolic diseases. LFC was quantified with two methods: MRS and magnetic resonance imaging (MRI). In contrast to previous studies with short accelerometer measurements, SB and PA were assessed with accelerometers continuously for one month to get a more comprehensive representation of daily behaviors. We also examined the associations between LFC and fitness, daily nutrient and energy intake, and common markers of cardiometabolic health. Lastly, we also evaluated the correlation and agreement between the two different LFC quantification methods.

## Methods

### Study design

This study used the baseline data of an intervention trial (Medical and physiological benefits of reduced sitting, ClinicalTrials.gov ID NCT03101228) performed at the Turku PET Centre, Turku, Finland between April 2017 and August 2019. All participants gave written informed consent. The study was conducted according to good clinical practice and the Declaration of Helsinki and approved by the Ethics Committee of the Hospital District of Southwest Finland (16/1810/2017).

### Participants

The participants were physically inactive, middle-aged adults with MetS^3^ who were recruited locally through bulletin boards and newspaper advertisements.The inclusion and exclusion criteria are described in Table 1.

**Table 1:** Inclusion and exclusion criteria.

Inclusion criteria	Exclusion criteria
(1) Age 40–65 years	(1) History of a cardiac event
(2) BMI 25–40 kg/m^2^	(2) Insulin or medically treated diabetes
(3) Physically inactive (less than 120 min of moderate-intensity exercise per week reported during screening and initial physical activity questionnaires)	(3) Any chronic disease or condition that could create a hazard to the subject safety, endanger the study procedures or interfere with the interpretation of study results
(4) Sitting time ≥ 10 h /day or 60% of accelerometer wear time (measured during screening)	(4) Abundant use of alcohol (according to national guidelines)
(5) Blood pressure < 160/100 mmHg	(5) Use of narcotics, smoking of tobacco or consuming snuff tobacco
(6) Fasting plasma glucose < 7.0 mmol/l	(6) Diagnosed depressive or bipolar disorder
(7) Fulfilment of the metabolic syndrome criteria (3), including three of the following symptoms Central obesity (WC ≥ 94 cm for men and ≥ 80 cm for women) Blood triglycerides ≥ 1.7 mmol/l HDL cholesterol < 1.0 mmol/l for men and < 1.3 mmol/l for women Systolic blood pressure ≥ 130 and/or diastolic blood pressure ≥ 85 mmHg Fasting glucose > 5.6 mmol/l	(7) Inability to understand written Finnish

### Measurements

#### Liver fat content

LFC was measured by MRS and MRI, based on two-point Dixon [2PD] method using a Philips 3 Tesla system (Ingenuity TF PET/MR) with a Q-Body coil. Because of the MRI scanner replacement during this study, MRS and MRI quantification of LFC of seven participants were conducted with Siemens Magnetom Skyra fit 3T MRI system (Siemens Healthcare, Erlangen, Germany) with Siemens Body 30 and 18 channel coils, and 32 channel Spine coil. A detailed description of the measurement is presented in the Supplementary Material (S1).

#### SB and PA measurements

SB and PA were measured for four weeks with validated hip-worn tri-axial accelerometers (UKK AM30, UKK-Institute, Tampere, Finland) and analysis methods. In short, the collected accelerometer data was analyzed in six-second epochs and SB (sitting and lying together), standing, light physical activity (LPA), moderate-to-vigorous physical activity (MVPA), steps, and breaks in SB were defined using validated mean amplitude deviation (MAD)^23^ and angle for posture estimation (APE) methods^24^. The following SB and PA variables were calculated: mean daily SB time, mean daily standing time, mean daily LPA time, mean daily MVPA time, and mean daily steps. For a valid data collection, daily wear time of 10–19 h and at least 4 days of valid measurements were required. Daily measurement time exceeding 19 h indicates that the participant has likely slept with the accelerometer and measurement hours exceeding the 19 h per day were substracted from the SB time.

#### Cardiorespiratory fitness

Maximal oxygen consumption (VO_2max_) measurements were conducted after the participants had passed a thorough physical examination and electrocardiographical measurements. VO_2max_ was determined by bicycle ergometry (eBike EL Ergometer + CASE v6.7, GE Medical Systems Information Technologies, Inc. Milwaukee, WI, USA) with direct respiratory gas measurements (Vyntus CPX, CareFusion, Yorba Linda, CA, USA). We also determined VO_2max_ per fat free mass (FFM) (ml/min/kg_FFM_), and maximal load (Wmax). A detailed description of the measurements is included in Supplementary Material (S1).

#### Whole-body insulin sensitivity and blood sampling

Whole-body insulin-stimulated glucose uptake (M-value) was measured and calculated with the gold standard method hyperinsulinemic-euglycemic clamp, as previously reported^25^. Venous blood samples were drawn after at least 10 h of fasting. Plasma glucose, plasma insulin, hemoglobin A_1c_ (HbA_1c_), plasma triglycerides, total cholesterol, LDL, HDL, alanine aminotransferase (ALT), aspartate aminotransferase (AST) and γ-glutamyltransferase (GGT) were determined according to methods described in the Supplementary Material (S1). Homeostatic model assessment of insulin resistance (HOMA-IR) was calculated using the formula: fasting glucose (mmol/l) × fasting insulin (mU/l)/22.5.

#### Nutrient intake

Nutrient intake was examined by 4-day food diaries (including one weekend day) and analyzed by a nutritionist with a computerized software (AivoDiet 2.2.0.1, Aivo, Turku) utilizing the Finnish Food Composition Database Fineli^26^. Participants were guided not to change their normal dietary habits during the study.

#### Body composition, anthropometry, blood pressure and resting heart rate

Participants could choose the measurement time by convenience. Validated^27^ air displacement plethysmography (the Bod Pod system, COSMED, Inc., Concord, CA, USA) with predicted thoracic gas volume was used to estimate body composition (body fat%, fat mass and fat-free mass) after fasting for at least four hours. Participants were advised not to exercise or take a shower beforehand on the day of the measurement. After emptying the bladder, participants entered the measurement chamber wearing a tight cap and underwear or swimming suit. Body weight, body height, waist circumference, blood pressure and resting heart rate were measured according to details reported in the Supplementary Material (S1).

### Statistical methods

The associations between LFC (dependent variable) and SB and PA measures, fitness, health markers and nutrient intake (independent variables) were examined with linear mixed models. Unpaired t-test was first used to compare sexes. All the models were adjusted for age, and because of a significant between-sex difference in LFC values, sex was also included as an explanatory variable in all the analyses (model 1). Body fat% was added to the linear model to adjust for confounding overweight or obesity (model 2). Normal distribution of the residuals was assessed by visual evaluation and Shapiro–Wilk test, and logarithmic transformations were used when necessary to fulfil the normal distribution assumption. Linear regression model, Tukey mean difference test and Bland–Altman analysis were used to analyze the correlation and the agreement between MRS and MRI (2PD); the results are reported in the Supplementary File (S2). Multicollinearity was controlled for with variance inflation factors, which were all below five indicating no multicollinearity issues. Missing data was handled by pairwise deletion. Power calculation for determining the sample size was done for the primary outcome (whole-body insulin sensitivity) of the sedentary behaviour reduction intervention trial (NCT03101228), from which baseline imaging measurements form the data for the current study. MRS-measured LFCs of three participants were missing due to image artifacts and MRS and MRI-measured LFCs of one participant were missing due to technical challenges with the scanner. VO_2max_ measures of two participants were missing because they interrupted the test before reaching exhaustion (knee pain or difficulties in breathing) and the results of one participant were lost due to technical difficulties. Fasting plasma glucose value of one participant and resting heart rate values of two participants were missing due to incomplete documentation. If not otherwise stated, data are expressed as mean and standard deviation (SD), standardized β coefficients and 95% CI values. The level of statistical significance was set at 5% (two-tailed). All analyses were carried out with the JMP pro 13.1 for Windows (SAS Institute Inc., Cary, NC, USA) and with GraphPad Prism version 5.01 for Windows (GraphPad Software, La Jolla California USA).

## Results

### Characteristics of the participants

Participants’ baseline characteristics are presented by sex in Table 2. Sixty-six percent of the participants were obese (BMI > 30 kg/m^2^) and 34% were overweight (BMI 25.0 to < 30). Men had significantly higher LFC, AST, HOMA-IR, and fasting insulin levels compared to women, while women had higher body fat% and HDL levels. Women also had slightly longer daily accelerometer wear time, standing time, daily standing%, LPA and LPA% and breaks in SB. On the other hand, men had higher daily SB% and VO_2max_ levels when compared to women.

**Table 2:** Characteristics of the study participants by sex. If not otherwise stated, the results are reported as mean (SD).

	Men	Women
n, (% of total)	19 (43)	25 (57)
Age, years	58 (6.0)	57 (7.3)
**Anthropometrics**		
BMI, kg/m^2^	31.8 (4.7)	32.5 (4.1)
Waist circumference, cm	115.2 (13.2)	108.4 (9.5)
Body fat %	37.5 (7.8)	48.1 (3.8)*
**Liver fat content**		
MRS, fat fraction %	5.1 (3.8)	2.6 (3.0)*
MRI, fat fraction %	11.9 (5.8)	8.0 (3.9)*
**Health measurements**		
Systolic blood pressure, mmHg	140 (14)	146 (13)
Diastolic blood pressure, mmHg	89 (9)	89 (6)
Blood pressure medication, n (%)	13 (68)	9 (36)
Cholesterol medication, n (%)	4 (21)	4 (16)
Resting heart rate, bpm	67 (6)	68 (10)
f-Glucose, mmol/l	5.9 (0.5)	5.7 (0.2)
f-Insulin, mU/l	16.2 (9.4)	10.4 (4.3)*
HbA_1c_, mmol/mol	37.8 (2.5)	36.8 (2.7)
HOMA-IR	4.3 (2.7)	2.7 (1.1)*
M-value, mg/kg/min	2.9 (2.8)	3.6 (2.1)
Triglycerides, mmol/l	1.4 (0.5)	1.3 (0.7)
Cholesterol, mmol/l	4.4 (0.7)	4.9 (1.1)
HDL, mmol/l	1.1 (0.3)	1.4 (0.3)**
LDL, mmol/l	2.9 (0.7)	3.2 (1.0)
ALT, U/l	36 (18)	27 (12)
AST, U/l	31 (12)	23 (5)*
GGT, U/l	34 (21)	25 (17)
**Accelerometry**		
Lying time, h/days	1.9 (0.9)	1.6 (0.5)
Sitting time, h/day	8.3 (1.1)	8.5 (1.0)
Sedentary time, h/day	10.3 (0.9)	10.1 (0.9)
Sedentary time, % of daily wear time	72 (7)	68 (5)*
Accelerometry, days	26 (2)	27 (3)
Wear time, h/day	14.3 (1.0)	14.9 (0.8)*
Breaks in SB, time/day	24 (5)	32 (8)**
Standing, h/day	1.5 (0.4)	2.0 (0.5)***
Standing, % of daily wear time	10.1 (2.8)	13.3 (3.2)**
Daily steps	5194 (2134)	4986 (1382)
LPA, h/day	1.6 (0.5)	1.9 (0.3)*
LPA, % of daily wear time	10.9 (3.2)	12.7 (2.1)*
MVPA, h/day	1.0 (0.4)	0.9 (0.2)
MVPA, % of daily wear time	6.9 (2.7)	6.2 (1.6)
PA, h/day	2.6 (0.7)	2.8 (0.4)
PA, % of daily wear time	17.8 (4.5)	18.9 (2.7)
**Cardiorespiratory fitness**		
VO_2max_, ml/min/kg	24.7 (5.6)	21.3 (3.4)*
**Nutrition**		
Total EI, kcal/day	1884.0 (377.8)	1742.5 (341.9)
Protein, % of daily EI	17.9 (2.9)	17.8 (2.8)
Carbohydrates, % of daily EI	39.7 (7.8)	40.7 (6.0)
Fat, % of daily EI	38.8 (6.2)	38.0 (5.2)
Alcohol, % of daily EI	1.7 (3.5)	1.4 (1.6)
SFA, % of daily EI	14.5 (3.3)	13.7 (2.4)
MUFA, % of daily EI	13.5 (3.7)	12.9 (2.0)
PUFA, % of daily EI	5.6 (1.5)	5.9 (1.2)
Saccharose, % of daily EI	7.3 (3.2)	8.3 (4.2)

*ALT* alanine aminotransferase, *AST* aspartate aminotransferase, *EI* energy intake, *GGT* γ-glutamyltransferase, *HbA*_*1c*_ hemoglobin A_1c,_, *HOMA-IR* homeostatic model assessment for insulin resistance, *f* fasting, *LPA* light physical activity, *MRI* magnetic resonance imaging, *MRS* magnetic resonance spectroscopy, *MUFA* monounsaturated fatty acids, M-value whole-body insulin sensitivity, *MVPA*  moderate to vigorous physical activity, *PA* physical activity (LPA + MVPA), *SB* sedentary behaviour (sitting + lying), *SFA* saturated fatty acids, *PUFA* polyunsaturated fatty acids, *VO*_*2max*_ maximal oxygen consumption.

Significant p-values; *p < 0.05, **p < 0.01, ***p < 0.001, vs men. Sex difference in t-test (or Fisher’s exact test, when applicable).

### Associations of body composition with SB and PA

The associations between body fat%, WC, BMI and SB, PA and fitness are presented in Table 3. When adjusted for age and sex, body fat% associated positively with lying time (h/day) and SB time (%/day), and negatively with standing (h/day), daily steps, MVPA, MVPA%, total PA and VO_2max_. WC associated positively with lying time and SB%, and negatively with standing, standing%, MVPA, MVPA%, total PA, daily steps and breaks in SB. BMI associated positively with lying time, and negatively with standing, MVPA, daily steps, breaks in SB and VO_2max_.

**Table 3:** Age -and sex -adjusted linear mixed regression estimates (standardized β coefficients (95% CI)) between body composition, sedentary behavior, physical activity and fitness.

	Body fat %		WC		BMI	
	β	p	β	p	β	p
Lying time, h/day	0.25 (0.01, 0.48)	**0.04**	0.39 (0.10, 0.68)	**0.009**	0.49 (0.2, 0.77)	**0.002**
Sitting time, h/day	− 0.12 (− 0.36, 0.11)	0.30	− 0.08 (− 0.38, 0.22)	0.59	− 0.21 (− 0.52, 0.1)	0.18
Sedentary time, h/day	0.05 (− 0.19, 0.29)	0.68	0.20 (− 0.10, 0.50)	0.18	0.13 (− 0.18, 0.45)	0.40
Sedentary time, % of daily wear time	0.27 (0.03, 0.51)	**0.03**	0.38 (0.08, 0.68)	**0.02**	0.32 (− 0.001, 0.64)	0.051
Breaks in SB, times/day	− 0.24 (0.50, − 0.02)	0.07	− 0.39 (− 0.72, − 0.07)	**0.02**	− 0.50 (− 0.83, − 0.18)	**0.003**
Standing, h/day	− 0.28 (− 0.54, − 0.02)	**0.03**	− 0.39 (− 0.71, − 0.06)	**0.02**	− 0.36 (− 0.70, − 0.01)	**0.04**
Standing, % of daily wear time	− 0.22 (− 0.48, 0.04)	0.68	− 0.35 (− 0.67, − 0.03)	**0.03**	− 0.30 (− 0.64, 0.04)	0.08
Steps, number/day	− 0.38 (− 0.59, − 0.16)	**0.001**	− 0.48 (− 0.76, − 0.20)	**0.001**	− 0.44 (− 0.74, − 0.15)	**0.004**
LPA, h/day	− 0.12 (− 0.38, 0.13)	0.33	− 0.12 (− 0.44, 0.21)	0.47	− 0.14 (− 0.48, 0.20)	0.41
LPA, % of daily wear time	− 0.08 (− 0.33, 0.17)	0.52	− 0.09 (− 0.41, 0.24)	0.59	− 0.09 (− 0.43, 0.24)	0.58
MVPA, h/day	− 0.33 (− 0.55, − 0.10)	**0.005**	− 0.42 (− 0.7, − 0.13)	**0.005**	− 0.35 (− 0.66, − 0.03)	**0.03**
MVPA, % of daily wear time	− 0.3 (− 0.53, − 0.06)	**0.01**	− 0.4 (− 0.69, − 0.11)	**0.008**	− 0.31 (− 0.63, 0.002)	0.052
PA, h/day	− 0.26 (− 0.49, − 0.03)	**0.03**	− 0.31 (− 0.6, − 0.01)	**0.04**	− 0.28 (− 0.59, 0.03)	0.074
PA, % of daily wear time	− 0.22 (− 0.45, 0.01)	0.06	− 0.28 (− 0.58, 0.01)	0.059	0.24 (− 0.55, 0.07)	0.13
VO_2_max, ml/min/kg	− 0.62 (− 0.84, − 0.40)	** < 0.0001**	− 0.65 (− 0.97, − 0.34)	**0.0002**	− 0.76 (− 1.07, − 0.45)	** < 0.0001**

*BMI* body mass index, *LPA* light physical activity; *MVPA* moderate-to-vigorous physical activity, *PA* physical activity (LPA and MVPA together), *SB* sedentary behaviour (sitting + lying), *VO*_*2max*_ maximal oxygen consumption, *WC* waist circumference.

Significant values are in bold.

### Associations of LFC with SB and PA

When adjusted for age and sex, LFC was not associated with any of the SB or PA variables (model 1, Table 4). Associations remained non-significant when body fat% was added to the model (model 2, Table 4). The associations of MRI-measured LFC with SB and PA are presented in the Supplementary File (S2).

**Table 4:** Age-, sex- and body fat % -adjusted linear mixed regression estimates (standardized β coefficients (95% CI)) between MRS-measured LFC, sedentary behavior and physical activity.

	LFC MRS^a^ (%)			
	Model 1		Model 2	
	β	p	β	p
Lying time, h/day	0.08 (− 0.25, 0.41)	0.62	0.01 (− 0.33, 0.30)	0.93
Sitting time, h/day	− 0.09 (− 0.41, 0.23)	0.58	− 0.09 (− 0.38, 0.22)	0.54
Sedentary time, h/day	− 0.04 (− 0.35, 0.28)	0.81	− 0.09 (− 0.40, 0.20)	0.55
Sedentary time, % of daily wear time	0.03 (− 0.31, 0.36)	0.88	− 0.14 (− 0.47, 0.22)	0.41
Breaks in SB, times/day	− 0.25 (− 0.61, 0.10)	0.15	− 0.17 (− 0.53, 0.15)	0.31
Standing, h/day	− 0.02 (− 0.39, 0.35)	0.92	0.08 (− 0.30, 0.42)	0.64
Standing, % of daily wear time	− 0.01 (− 0.37, 0.34)	0.93	0.06 (0.29, 0.39)	0.72
Steps, number/day	− 0.29 (− 0.61, 0.02)	0.07	− 0.11 (− 0.52, 0.25)	0.56
LPA, h/day	0.17 (− 0.20, 0.54)	0.35	0.26 (− 0.09, 0.60)	0.13
LPA, % of daily wear time	0.17 (− 0.18, 0.51)	0.34	0.23 (− 0.09, 0.56)	0.15
MVPA, h/day	− 0.22 (− 0.54, 0.11)	0.18	− 0.03 (− 0.42, 0.31)	0.88
MVPA, % of daily wear time	− 0.21 (− 0.54, 0.12)	0.21	− 0.02 (− 0.41, 0.32)	0.90
PA, h/day	− 0.04 (− 0.37, 0.30)	0.83	0.17 (− 0.20, 0.50)	0.32
PA, % of daily wear time	− 0.02 (− 0.35, 0.30)	0.88	0.15 (− 0.18, 0.47)	0.34

Model 1 adjusted for age and sex.

Model 2 adjusted for age, sex and body fat %

*LFC* liver fat content, *LPA* light physical activity, *MUFA* monounsaturated fatty acids, *MRS* magnetic resonance spectroscopy, *MVPA* moderate to vigorous physical activity, *PA* physical activity (LPA and MVPA together), *SB* sedentary behaviour (sitting and lying).

^a^log10 transformed variables.

### Associations of LFC with fitness and nutrient intake

In the sex- and age-adjusted model LFC was not associated with VO_2 max_ (ml/min/kg) (model 1, Table 5), and when body fat% was included in the model, the association remained non-significant (model 2, Table 5). Also, when fitness was expressed as VO_2_max (ml/min/kg_FFM_) or Wmax, none of the associations were significant (model 1–2, Table 5). In the sex- and age-adjusted model LFC was not associated with any of the nutrient intake variables expressed as % of daily energy intake (model 1, Table 5). When body fat% was added to the model, all other associations were non-significant except for the association between LFC and protein intake (model 2, Table 5). The associations of MRI-measured LFC with fitness and nutrient intake are presented in the Supplementary File (S2).

**Table 5:** Age-, sex- and body fat %-adjusted linear mixed regression estimates (standardized β coefficients (95% CI)) between MRS-measured LFC, fitness and dietary intake.

	LFC MRS^a^ (%)			
	Model 1		Model 2	
	β	p	β	p
VO_2max_, ml/min/kg	− 0.39 (− 0.80, 0.03)	0.07	− 0.07 (− 0.58, 0.44)	0.79
VO_2max_, ml/min/kg_FFM_	− 0.10 (− 0.48, 0.28)	0.61	− 0.13 (− 0.48, 0.22)	0.45
Maximal load, W	− 0.14 (− 0.57, 0.30)	0.53	− 0.02 (− 0.44, 0.40)	0.93
Total EI, kcal/day	0.14 (− 0.19, 0.48)	0.40	0.14 (− 0.17, 0.45)	0.36
Protein, % of daily EI	− 0.25 (− 0.55, 0.06)	0.11	− 0.31 (− 0.59, − 0.03)	**0.03**
Carbohydrates, % of daily EI	− 0.05 (− 0.37, 0.28)	0.77	0.08 (− 0.24, 0.41)	0.60
Fat, % of daily EI	− 0.05 (− 0.15, 0.50)	0.28	0.06 (− 0.26, 0.39)	0.70
Alcohol, % of daily EI	− 0.02 (− 0.34, 0.30)	0.89	0.01 (− 0.29, 0.32)	0.92
SFA, % of daily EI	0.05 (− 0.28, 039)	0.74	0.10 (− 0.22, 0.41)	0.54
MUFA, % of daily EI	0.16 (− 0.15, 0.48)	0.31	0.01 (− 0.32, 0.33)	0.97
PUFA, % of daily EI	0.1 (− 0.22, 0.41)	0.53	− 0.03 (− 0.35, 0.28)	0.83
Saccharose, % of daily EI	0.11 (− 0.20, 0.43)	0.47	0.11 (− 0.13, 0.46)	0.27

Model 1 adjusted for age and sex.

Model 2 adjusted for age, sex and body fat %

*EI* energy intake, *FFM* fat free mass, *LFC* liver fat content, *MUFA* monounsaturated fatty acids, *MRI* magnetic resonance imaging, *MRS* magnetic resonance spectroscopy, *PUFA* polyunsaturated fatty acids, *SFA* saturated fatty acids, *VO*_*2max*_ maximal oxygen consumption.

^a^log10 transformed variables.

Significant values are in bold.

Additionally, we tested the associations between LFC (measured by MRS and MRI) and the daily intake of protein, carbohydrates, fat, alcohol, saturated fatty acids (SFA), monounsaturated fatty acids (MUFA), polyunsaturated fatty acids (PUFA), saccharose and fiber measured in grams. MRS-measured LFC was not associated with any of the nutrient variables, when adjusted for sex and age, nor when further adjusted for body fat % (data not shown). MRI-measured LFC was associated with MUFA (g) (β = 0.35, 95% CI [0.05, 0.64], p = 0.02). However, when body fat % was added to the model, the association turned non-significant (β = 0.22, 95% CI [− 0.007, 0.50], p = 0.13).

### Associations of LFC with body adiposity and other health markers

When adjusted for age and sex, LFC associated positively with body fat%, BMI, WC, triglycerides, ALT, fasting insulin, HOMA-IR, M-value and HbA1c (model 1, Table 6). After further adjustment for body fat%, LFC remained positively associated with WC, M-value, HbA1c, triglycerides and ALT, and the association between LFC and GGT turned significant. On the other hand, the associations between LFC and fasting insulin and HOMA-IR turned non-significant (model 2, Table 6). The associations between the MRI-measured LFC with body adiposity and other health markers are presented in the Supplementary File (S2).

**Table 6:** Age-, sex- and body fat %-adjusted linear mixed regression estimates (standardized β coefficients (95% CI)) between MRS-measured LFC, body composition and cardiometabolic risk factors.

	LFC MRS^a^ (%)			
	Model 1		Model 2	
	β	p	β	p
Body fat, %	0.50 (0.10, 0.89)	**0.02**		
Waist, cm	0.58 (0.31, 0.85)	**0.0001**	0.55 (0.20, 0.91)	**0.003**
BMI, kg/m^2^	0.34 (0.05, 0.64)	**0.03**	0.17 (− 0.23, 0.57)	0.40
SBP, mmHg	− 0.08 (− 0.41, 0.26)	0.64	− 0.11 (− 0.42, 0.20)	0.49
DBP, mmHg	0.13 (− 0.18, 0.45)	0.40	0.08 (− 0.23, 0.38)	0.61
Resting heart rate, bpm	− 0.21 (− 0.52, 0.11)	0.19	− 0.19 (− 0.47, 0.09)	0.18
BP medication	0.08 (− 0.26, 0.42)	0.65	0.02 (− 0.31, 0.34)	0.90
Cholesterol medication	− 0.06 (− 0.38, 0.26)	0.70	0.02 (− 0.29, 0.32)	0.91
f-Glucose, mmol/l	− 0.11 (− 0.44, 0.21)	0.49	− 0.13 (− 0.43, 0.17)	0.40
f-Insulin, mU/l	0.41 (0.09, 0.72)	**0.01**	0.31 (− 0.02, 0.63)	**0.06**
HOMA-IR	0.38 (0.06, 0.70)	**0.02**	0.28 (− 0.004, 0.60)	0.084
M-value, mg/kg/min	− 0.43 (− 0.72, − 0.14)	**0.005**	− 0.34 (− 0.67, − 0.01)	**0.047**
HbA1c, mmol/mol	0.54 (0.27, 0.81)	**0.0003**	0.48 (0.20, 0.75)	**0.001**
Triglycerides, mmol/l	0.38 (0.09, 0.67)	**0.01**	0.38 (0.11, 0.64)	**0.006**
Cholesterol, mmol/l	0.22 (− 0.10, 0.53)	0.18	0.22 (− 0.07, 0.52)	0.14
HDL, mmol/l	− 0.18 (− 0.57, 0.17)	0.29	− 0.18 (− 0.52, 0.17)	0.31
LDL, mmol/l	0.18 (− 0.13, 0.49)	0.25	0.18 (− 0.12, 0.47)	0.23
ALT, U/l	0.50 (0.23, 0.78)	**0.0007**	0.46 (0.20, 0.73)	**0.001**
AST, U/l	0.24 (− 0.10, 0.59)	0.16	0.32 (− 0.003, 0.64)	0.052
GGT, U/l	0.30 (− 0.01, 0.60)	0.054	0.29 (0.01, 0.57)	**0.04**

Model 1 adjusted for age and sex.

Model 2 adjusted for age, sex and body fat %

*ALT* alanine aminotransferase, *AST* aspartate aminotransferase, *BP* blood pressure, *DBP* diastolic blood pressure, *f* fasting, *GGT* γ-glutamyltransferase, *HbA*_*1c*_ hemoglobin A_1c_, *HOMA-IR* homeostatic model assessment for insulin resistance, *LFC* liver fat content, *MRS* magnetic resonance spectroscopy, *M-value* whole-body insulin sensitivity, *SBP* systolic blood pressure.

^a^log10 transformed variables.

Significant values are in bold.

## Discussion

In the present study, we found that LFC is not associated with accelerometer-measured SB, habitual PA or fitness in sedentary adults with MetS. Additionally, we found that LFC is associated with body fat%, BMI and WC, and also with other health risk markers (insulin resistance, fasting triglycerides and circulating liver enzymes) independent of body adiposity. We also demonstrated that body composition (body fat%, BMI and WC) was positively associated with daily SB and negatively with habitual PA. Thus, our results indicate that body adiposity is a key regulator of LFC, but LFC is also independently clustered with other cardiometabolic risk factors. We also detected that LFC was negatively associated with the energy intake from protein, which might refer that replacing some of the daily carbohydrates and/or fat with protein sources might improve liver health. Our study gives new insights to the associations between LFC and accelerometer-measured SB and PA, fitness and nutrient intake in adults with MetS. Additionally, our study shows the compatibility of MRS and MRI (2PD) for measuring LFC.

### Associations of LFC with SB and PA

LFC was not associated with SB, standing or habitual PA performed at different intensities. When adjusted for sex and age, only the association between MRI-measured LFC and daily steps was significant. However, when body fat% was added to the model the association was non-significant, suggesting that the association between LFC and steps is mediated by body adiposity. Although with a larger number of participants, the majority of the previous studies investigating associations between LFC and SB and PA have used self-reports to determine SB and PA. Most have reported a positive association between LFC and SB^15,16^ and a negative association between LFC and PA^12–14,17^. Additionally, a stronger association between LFC and PA has been shown with increased amount and intensity of PA^12–14^. However, in one previous study the association between LFC and PA was attenuated when WC was added to the model^17^. SB has also been suggested to be an independent predictor of NAFLD based on the data from the National Health and Nutrition Examination Survey (NHANES) 2007–2016^28^. The NHANES 2007–2016 study also indicated that increasing habitual and transportation-related PA would lower the risk of NAFLD in a dose-dependent manner^28^. Although the study had a very large nationally representative cohort of US adults, SB was likely underestimated and PA overestimated since SB and PA levels were based on self-reports^18^.

On the other hand, the few studies with accelerometer-measured PA have shown mixed results. In contrast to our findings, a positive association between the measured SB and MRS-measured liver fat% was found in 98 habitually active young—middle aged adults with or without MetS, when adjusted for age and BMI^19^. Another study with sixty-six adults found that more time spent in SB was associated with higher liver fat% in individuals at high risk of developing metabolic diseases such as type 2 diabetes^20^. However, there is also evidence supporting our findings. For example, in adults with overweight or obesity liver fat was not associated with the measured habitual PA or SB^21^.

Differences in the results might originate from differences in study populations, methods, and genetic variation in fitness levels^29^. Most of the previous studies determined LFC by ultrasound or fatty liver index, instead of the gold standard assessment methods liver biopsy and MRS, which may affect the results. An important difference in the methods pertains to the variation in data collection time between the studies. In all previous studies the accelerometer-measured PA was collected during 4–7 days, whereas in our study the mean data collection was 26 days. This is likely to provide a more comprehensive representation of daily behaviors compared to short measurement periods. In addition, the placement of the accelerometer can also have an impact on the results. Majority of the previous studies used wrist-worn accelerometers to measure SB and PA^19,21^, whereas, we used hip-worn accelerometers. With this placement the device is closer to the center of mass of the body, and thus detects more accurately body motion and postural differences^30^.

Lastly, it seems that the intensity and the duration of PA might be they key factors to reveal any significant associations between LFC and PA. For example, a recent meta-analysis showed that high and moderate levels of PA are associated with a reduced risk of NAFLD, and that the risk is reduced in a dose-dependent manner. The meta-analysis also suggested that PA amount above the recommended minimum of 150 min of moderate- or 75 min of vigorous-intensity activity might be required to achieve a considerable reduction in NAFLD risk^31^. Thus, the major reason for not finding any associations between LFC and PA in our study might be the inactive and sedentary population that was used. It is possible that there was not enough variation in PA levels in our homogenous study group. Thus, our data suggests that neither SB nor habitual PA is independently associated with LFC in inactive adults with metabolic syndrome, and a higher amount and/or intensity of PA might be needed to improve LFC.

### Associations of LFC with fitness and nutrition

When adjusted for sex and age, MRI-measured LFC was significantly associated with VO_2max_. However, when body fat % was added to the model the associations were attenuated, regardless of the measurement method of LFC. Similar results with same statistical adjustments have been reported previously^32^. Our finding together with previous evidence suggests that fitness is not independently associated with LFC, and the primary regulator of LFC is the overall adiposity, and thus reducing excess body fat would be the key factor to impact LFC.

We also found that, when adjusted for sex, age and body fat%, MRS- and MRI-measured LFC was negatively associated with daily protein intake (% of total energy intake). This is in line with a recent review^33^ that suggests that a diet high in protein, particularly that of plant-based origin, and a low content of carbohydrates and sugars would be one strategy to improve LFC and insulin sensitivity.

We did not find any significant associations between LFC and intakes of carbohydrates, sugars or saturated or unsaturated fatty acids. The reason for this could be that there was not enough variation in the nutrient intake in our study participants with overweight and obesity. It has been shown that fat and carbohydrates can have different influence in liver fat accumulation, and the effects may differ based on the type of the macronutrient^34,35^. Saturated fatty acids and fructose have been found to induce the greatest increases in intrahepatic triglycerides, and on the other hand, unsaturated fatty acids have been found to have beneficial effects on liver health^34–36^. Recent meta-analysis showed that replacing saturated fatty acids with unsaturated fatty acids leads to decrease in liver fat content^35^. Additionally, previous studies indicate that diets with low carbohydrate content are beneficial for subjects with NAFLD^37^. Thus, it seems that replacing some of the daily carbohydrates and/or fats with protein sources might associate with healthier LFC in adults at increased risk of cardiometabolic diseases. For summary, previous studies shows that both the quality and quantity of different macronutrients play important role in liver fat accumulation. Nevertheless, it seems that the total daily calories rather than different proportions of macronutrients may be more important factor for the liver fat content^34,38^.

### Associations of LFC with general health markers

In our study, LFC was positively associated with markers of obesity (body fat%, BMI and WC). Similarly, previous studies have also shown a strong association between fatty liver and obesity^39–41^. Moreover, high BMI increases NAFLD risk in a dose-dependent manner^40^. In the current study LFC was also positively associated with insulin resistance markers such as fasting insulin, M-value, HOMA-IR and HbA1c, when adjusted for sex and age. However, only the associations between LFC and M-value and HbA1c remained significant when body fat% was added to the model. Similar associations between LFC and risk factors related to glucose metabolism have been reported earlier^42,43^. For example, fatty liver index has been positively associated with insulin resistance, coronary heart disease and atherosclerosis^42^.

We showed that LFC is positively associated with fasting plasma triglycerides. Fasting triglycerides have also previously been shown to strongly associate with NAFLD, and elevated triglyceride levels in the blood have been indicated as markers of high LFC in adults with or without NAFLD^33^. Additionally, we found that LFC was positively associated with circulating liver enzymes (ALT, AST and GGT). This was expected, since serum liver enzyme levels are commonly used to detect liver diseases. However, these enzymes are not always elevated in NAFLD^44^. Diagnosed only with blood sampling, the true prevalence of NAFLD might be significantly underestimated. To conclude, our results build on the existing evidence and support the notion that obesity and other cardiometabolic risk factors (e.g., insulin resistance, elevated blood lipids, and elevated liver enzyme levels) are closely associated with LFC.

### Correlation and agreement between MRS and MRI

In the present study we also tested the correlation and agreement between two LFC-quantification methods: MRS and MRI (2PD). Our results show that MRI correlated strongly with MRS (S2, Fig. 1). However, when we tested the agreement between these two methods (S2, Fig. 2), the mean difference was statistically significant. The reason for this is that MRI systematically gave higher (over 2.5 times greater) LFC-values than MRS. Previously liver biopsy has been shown to give over two times greater LFC-values than MR-techniques^45^. Liver biopsy is the most accurate method to quantify liver fat^46^, however, it is not always the most feasible method to use due to its invasive nature. Both MRS and MRI have been used to quantify LFC in clinical practice, but MRS has appeared to be a more accurate method when compared to other non-invasive methods^47^. However, MRS also has some limitations, e.g., LFC can be misestimated due to the small size of the measured liver section, especially in case of heterogenous steatosis. It is also more time-consuming and challencing to perform compared to MRI^45^. Further studies are warranted to determine which of these two methods (MRS or MRI [2PD]) gives the most accurate results to measure LFC, when compared to liver biopsy. This is important in terms of the correct NAFLD diagnosis, because possible underestimation of LFC with MRS might leave underlying NAFLD undetected.

### Strengths and limitations

The major strengths of our study are the methods that we used to quantify liver fat and SB and PA. LFC was measured with two different methods: MRI (2PD) and MRS, which is considered the golden standard method to non-invasively quantify liver fat^47^. We used validated algorithms and hip-worn accelerometers to measure SB and PA^23,24^. The placement of the accelerometer may provide more accurate representation of motion and postural differences than wrist-worn accelerometers^48^ used in previous investigations^19,21^. Additionally, one of the key strengths is the duration of the accelerometer data collection, which was considerably longer (26 days), compared to previous studies^19–21^, and thus can give more robust estimation of individual habitual PA and SB.

This study also has some limitations. Due to the MRI scanner replacement during the study, MRS and MRI quantification of LFC of seven participants were conducted with a different scanner, which might affect the results. Also, our sample size was relatively small, and power calculation was done only for longitudinal analysis. However, the used methods (MR, accelerometry as well as direct respiratory gas measurements in fitness test) are very sensitive for detecting small variations in the outcomes and therefore can be used with relatively small study samples. Lastly, very intensive studies for both study personnel and study participants were conducted. Additionally, we included only physically inactive sedentary participants. The results might have been different if we had included participants with more variation in their SB and habitual PA. However, previous evidence suggests that the intensity of PA may play an important role, and the amount of habitual PA by itself may not be strong enough stimulus to have a positive effect on LFC. It is also possible that the participants have either underestimated food intake or changed their eating behavior when filling the food diary. However, at the group level food diaries yield reliable information on the intake of energy yielding nutrients. More detailed analyses about the protein sources (animal or plant-based) would also have given more insights into the association between LFC and daily protein intake. However, it was beyond the scope of this study and can thus be considered a limitation.

## Conclusions

In this study based on a one-month accelerometer-measurement of SB an PA, LFC was not associated with SB or habitual daily PA in sedentary inactive adults with MetS. However, we found positive associations between LFC and obesity markers (body fat%, BMI, WC), suggesting that weight loss especially from the abdominal area, is likely the primary method to reduce fat in the liver. We also showed that body composition (body fat%, BMI and WC) was positively associated with daily SB and negatively with habitual PA. Thus, SB and habitual PA may not be the main regulators of LFC, but they may indirectly affect liver health through their effects on body composition. Additionally, the negative association between LFC and daily protein intake suggest that replacing some of the carbohydrates and/or fat with protein sources in diet might associate with healthier LFC. Future studies should aim to assess the relationship between LFC and SB, habitual PA, fitness, as well as nutrient intake, in longitudinal and experimental settings, which may show causal relations.

## Supplementary Information

Supplementary Information 1.

Supplementary Information 2.

## Data Availability

The datasets generated during the current study are available from the corresponding author on reasonable request.
