# Supplementary material for: Relationship between liver fat content and lifestyle factors in adults with metabolic syndrome
Source: Sci Rep. 2022 Oct 19;12:17428. doi: 10.1038/s41598-022-22361-3 (PMC9581946; doi:10.1038/s41598-022-22361-3)
Supplement: Supplementary file 1 — Supplementary Information 1. [file 41598_2022_22361_MOESM1_ESM.docx]

**Relationship between liver fat content and lifestyle factors in adults with metabolic syndrome**

Saara Laine^1^, Tanja Sjöros^1^, Taru Garthwaite^1^, Maria Saarenhovi^1,2^, Petri Kallio^2,6^, Eliisa Löyttyniemi^3^, Henri Vähä-Ypyä^4^, Harri Sievänen^4^, Tommi Vasankari^4,8^, Kirsi Laitinen^5^, Noora Houttu^5^, Ekaterina Saukko^9^, Juhani Knuuti^1^, Virva Saunavaara^1,7^ and Ilkka H.A Heinonen^1,10^

1. Turku PET Centre, University of Turku and Turku University Hospital, Turku, Finland
2. Department of Clinical Physiology and Nuclear Medicine, University of Turku and Turku University Hospital, Turku, Finland
3. Department of Biostatistics, University of Turku, Turku, Finland
4. The UKK institute for Health Promotion Research, Tampere, Finland
5. Institute of Biomedicine, University of Turku, Turku, Finland
6. Paavo Nurmi Center, Turku, Finland
7. Department of Medical Physics, Division of Medical Imaging, Turku University Hospital, Finland
8. Faculty of Medicine and Health Technology, Tampere University, Tampere, Finland
9. Department of Radiology, Turku University Hospital, Turku, Finland
10. Rydberg Laboratory of Applied Sciences, University of Halmstad, Halmstad, Sweden

**Supplementary material 1**

**Methods**

***Liver fat content***

Liver fat content (LFC) was measured by magnetic resonance spectroscopy (MRS) and by magnetig resonance imaging (MRI), based two-point Dixon [2PD] method using a Philips 3 Tesla system (Ingenuity TF PET/MR) with a Q-Body coil. The spectra were acquired using stimulated echo acquisition mode (STEAM) 1H MRS with parameters: repetition time (TR)/echo time (TE)/mixing time (TM) = 2000/11/17 ms, 4 averages, 2048 samples, spectral bandwidth 2000 Hz, and acquisition volume 20 x 20 x 30 mm3. Data was acquired during 12 breath-holds. Water saturation was done with chemical shift selective (CHESS) with 50 Hz bandwidth. Duration of the scan was 3:12.0. The 3D T1-fast field echo sequence was acquired in the axial plane with parameters: TR/TE1/TE2 = 2.8 / 0.81 / 1.8 ms, flip angle 10°, FOV 510 mm x 510 mm, imaging matrix 188 x 188. Data was reconstructed to voxel size 2.13 x 2.13 x 4 mm3. Respiratory gating was used in the thorax – upper abdomen area.

MRS and MRI quantification of LFC conducted with Siemens Magnetom Skyra fit 3 T MRI system (Siemens Healthcare, Erlangen, Germany) with Siemens Body 30 and 18 channel coils, and 32 channel Spine coil.The spectra were acquired with point resolved spectroscopy (PRESS) 1H MRS with parameters TR/TE = 4000/30 ms, averages 32, 1024 samples, acquisition volume 20 x 20 x 20 mm3. Respiratory motion was controlled using navigator. Water saturation was done with 35 Hz bandwidth. Duration of the scan was 3:10. The 3D gradient echo volumetric interpolated breath-hold examination (VIBE Dixon) sequence was acquired in the axial plane with parameters: TR/TE1/TE2 = 3.97 / 1.23 / 2.46, flip angle 9°, and voxel size 2 x 2 x 2 mm3. Breath holds were used in the thorax – upper abdomen area.Controlled aliasing in parallel imaging results in higher acceleration (CAIPIRINHA) was used. Water signal and fat signal images were used to calculate the fat fraction map (1) from which the LFC was determined; MRI image was used as an anatomical reference.

***Image analysis***

LC Model (Version 6.3-0C) was used to quantify liver fat with ‘liver-4’ as spectrum type. Lipid signals 1.6 ppm, 1.3 ppm and 0.9 were used. The fat and water signals were corrected due to difference in T2 decay (2,3) and molar concentrations of 1H nuclei in fat and water as reported before (4,5). Liver fat content was defined as fat in relation to the total weight of liver tissue (2).

MRI images were analyzed using Carimas software version 2.10 (http://turkupetcentre.fi/). Four representative three-dimensional regions of interest (ROIs) were drawn manually on the sections of the liver (left lateral and medial section, right anterior and posterior section) avoiding the main portal veins. The results were volume corrected with the following formula: mean volume of one section x (total volume (mm3) of one section / total volume of all sections).

***Cardiorespiratory fitness***

Exercise work load was started at 25 W and increased by 25 W every three minutes until exhaustion. Participants were instructed to maintain a pace of 60-65 rpm throughout the test. Blood pressure and perceived exertion on Borg scale (6) were measured one minute after each increase in work load. Maximal oxygen consumption (VO_2max_) was determined if one criterion was met:  respiratory exchange ratio > 1.0, plateau in VO2, or heart rate within ±10 bpm of the age-predicted maximum. VO_2max_ was defined as the highest one-minute average in ml/min/kg.

***Blood sampling***

Plasma glucose was determined by enzymatic reference method with hexokinase GLUC3 and plasma insulin was determined by electrochemiluminescence immunoassay (Cobas 8000 e801, Roche Diagnostics GmbH, Mannheim, Germany). Hemoglobin A_1c_ (HbA_1c_) was determined by turbidimetric inhibition immunoassay (Cobas 6000 c501, Roche Diagnostics GmbH, Mannheim, Germany). Plasma triglycerides, total cholesterol, low-density lipoprotein (LDL) and high-density lipoprotein (HDL) cholesterol by enzymatic colorimetric tests (Cobas 8000 c702, Roche Diagnostics GmbH, Mannheim, Germany). Alanine aminotransferase (ALT) and aspartate aminotransferase (AST) were determined by photometric IFCC (International Federation of Clinical Chemistry) method (Cobas 8000 c702 and c 502 Analyzer, Roche Diagnostics GmbH, Mannheim, Germany), and γ-glutamyltransferase (GGT) by enzymatic colorimetric tests and assay (Cobas 8000 c702, Roche Diagnostics GmbH, Mannheim, Germany). All the samples were analysed at the Turku University Hospital Laboratory.

***Anthropometry, blood pressure and resting heart rate***

Body weight was measured by a scale (Seca 797, Vogel & Halke, Hamburg, Germany) in light clothing and body height barefooted with a wall-mounted stadiometer. Body mass index (BMI) was calculated from the measured weight and height in kg/m^2^. Waist circumference (WC) was measured with a flexible measuring tape midline between the iliac crest and the lowest rib, and the measurement was repeated twice or until the same measure was obtained twice. Blood pressure and resting heart rate were measured using a digital blood pressure monitor (Apteq AE701f, Rossmax International LtD, Taipei, Taiwan) in a seated position after at least 5 min of sitting. The mean of 2-3 measurements was used as the outcome measure. The anthropometric variables were measured under standard conditions. All the measurements were performed by the same researcher to ensure standardized measurements.

1. Bray TJ, Chouhan MD, Punwani S, Bainbridge A, Hall-Craggs MA. Fat fraction mapping using magnetic resonance imaging: insight into pathophysiology. British journal of radiology. 2018;91(1089):20170344.

2. Thomsen C, Becker U, Winkler K, Christoffersen P, Jensen M, Henriksen O. Quantification of liver fat using magnetic resonance spectroscopy. Magnetic resonance imaging. 1994;12(3):487–95.

3. Weis J, Kullberg J, Ahlström H. Multiple breath‐hold proton spectroscopy of human liver at 3T: Relaxation times and concentrations of glycogen, choline, and lipids. Journal of magnetic resonance imaging. 2018;47(2):410–7.

4. Rigazio S, Lehto H-R, Tuunanen H, Nagren K, Kankaanpaa M, Simi C, ym. The lowering of hepatic fatty acid uptake improves liver function and insulin sensitivity without affecting hepatic fat content in humans. American journal of physiology: endocrinology and metabolism. 2008;295(2):413.

5. Szczepaniak LS, Babcock EE, Schick F, Dobbins RL, Garg A, Burns DK, ym. Measurement of intracellular triglyceride stores by H spectroscopy: validation in vivo. American Journal of Physiology - Endocrinology And Metabolism. 1999;276(5):977–89.

6. Borg GA. Psychophysical bases of perceived exertion. Medicine and science in sports and exercise. 1982;14(5):377–81.
