# Supplementary material for: Relationship between liver fat content and lifestyle factors in adults with metabolic syndrome
Source: Sci Rep. 2022 Oct 19;12:17428. doi: 10.1038/s41598-022-22361-3 (PMC9581946; doi:10.1038/s41598-022-22361-3)
Supplement: Supplementary file 2 — Supplementary Information 2. [file 41598_2022_22361_MOESM2_ESM.docx]

**Relationship between liver fat content and lifestyle factors in adults with metabolic syndrome**

Saara Laine^1^, Tanja Sjöros^1^, Taru Garthwaite^1^, Maria Saarenhovi^1,2^, Petri Kallio^2,6^, Eliisa Löyttyniemi^3^, Henri Vähä-Ypyä^4^, Harri Sievänen^4^, Tommi Vasankari^4,8^, Kirsi Laitinen^5^, Noora Houttu^5^, Ekaterina Saukko^9^, Juhani Knuuti^1^, Virva Saunavaara^1,7^ and Ilkka H.A Heinonen^1,10^

1. Turku PET Centre, University of Turku and Turku University Hospital, Turku, Finland
2. Department of Clinical Physiology and Nuclear Medicine, University of Turku and Turku University Hospital, Turku, Finland
3. Department of Biostatistics, University of Turku, Turku, Finland
4. The UKK institute for Health Promotion Research, Tampere, Finland
5. Institute of Biomedicine, University of Turku, Turku, Finland
6. Paavo Nurmi Center, Turku, Finland
7. Department of Medical Physics, Division of Medical Imaging, Turku University Hospital, Finland
8. Faculty of Medicine and Health Technology, Tampere University, Tampere, Finland
9. Department of Radiology, Turku University Hospital, Turku, Finland
10. Rydberg Laboratory of Applied Sciences, University of Halmstad, Halmstad, Sweden

**Supplementary material 2**

**Results**

***Correlation and agreement between MRS and MRI (2PD)***

Magnetic resonance spectroscopy (MRS) measured liver fat content (LFC) correlated strongly with magnetic resonance imaging (MRI [2PD]) measured LFC (r = 0.76, 95% CI [0.59 – 0.87], p<0.0001) **(Figure 1)**. However, when testing the agreement between these two methods the mean difference was statistically significant (MD 5.81, 95 % CI [4.78 – 6.84], p<0.001). Bland–Altman plot illustrates the difference between MRS-measured and MRI-measured LFC **(Figure 2)**.

***Associations of liver fat content with sedentary behaviour and physical activity***

The examination of associations of MRI-measured LFC with sedentary behaviour (SB) and physical activity (PA) showed similar results to MRS-measured LFC. The only difference was that in the age- and sex-adjusted model, MRI-measured LFC was negatively associated with daily steps **(model 1, table 1)**. However, this association turned non-significant when body fat % was added to the model **(model 2, table 1).**

***Associations of liver fat content with fitness and nutrient intake***

In the sex- and age-adjusted model a negative association between MRI-measured LFC and maximal oxygen consumption (VO_2 max,_ ml/min/kg) was observed **(model 1, table 2)**. When body fat-% was included in the model, the association between LFC and VO_2 max_ (ml/min/kg) turned non-significant **(model 2, table 2)**. When fitness was expressed as VO_2_max (ml/min/kg_FFM_) or Wmax, none of the associations were significant **(model 1-2, table 2)**.

When examining the age- and sex-adjusted associations between LFC and nutrient intake variables expressed as % of daily energy intake, a significant association was observed between the MRI-measured LFC and monounsaturated fatty acids (MUFA) **(model 1, table 2)**. When body fat-% was added to the model, all other associations were non-significant except for the association between MRI-measured LFC and protein intake **(model 2, table 2).**

***Associations of liver fat content with body adiposity and other health markers***

The majority of the associations between the MRI-measured LFC with body adiposity and other health markers showed similar results than the MRS-measured LFC **(model 1-2, table 3)**. Only in the age- and sex-adjusted model the association between LFC and BMI was not statistically significant and the association between AST turned significant. When body fat % was added to the model the association between LFC and WC and GGT turned non-significant, while the association between AST turned significant **(model 2, table 3)**.


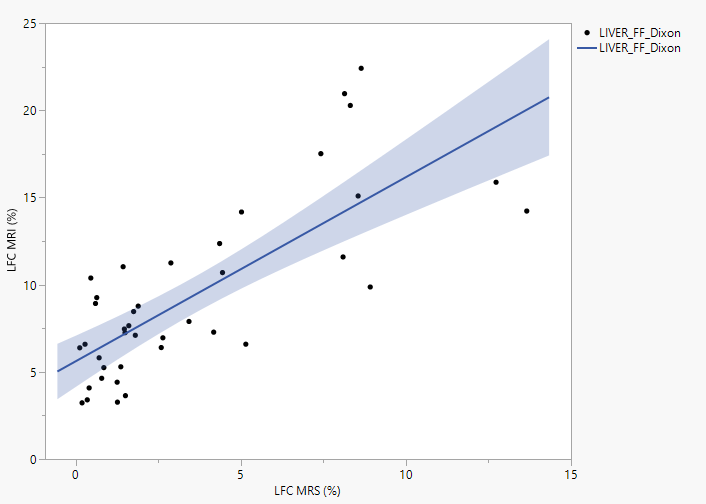


**Figure 1.** *Linear regression plot showing a positive correlation (r = 0.76, 95% CI [0.59 – 0.87], p<0.0001) between magnetic resonance spectroscopy (MRS) measured liver fat content (LFC) and magnetic resonance imaging (MRI, two-point Dixon method) measured LFC.*

**Figure 2.** *Result of Bland-Altman analysis. Solid lines delineate 95% CIs, and dashed lines show central bias. MRS = magnetic resonance spectroscopy, MRI = magnetic resonance imaging, 2PD = 2-point Dixon method.*

| **Table 1. Age-, sex- and body fat % -adjusted linear mixed regression estimates (standardized β coefficients (95% CI)) between MRI-measured LFC, sedentary behavior and physical activity.** | | | | |
| --- | --- | --- | --- | --- |
|  | **LFC MRI (%)** | | | |
|  | **Model 1** | | **Model 2** | |
|  | **β** | **p** | **β** | **p** |
| Lying time, h/day | 0.03  (-0.28, 0.34) | 0.86 | -0.13  (-0.41, 0.15) | 0.35 |
| Sitting time, h/day | -0.03  (-0.34, 0.27) | 0.82 | 0.05  (-0.22, 0.32) | 0.73 |
| Sedentary time, h/day | -0.02  (-0.32, 0.28) | 0.90 | -0.04  (-0.3, 0.23) | 0.77 |
| Sedentary time, % of daily wear time | 0.10  (-0.21, 0.42) | 0.51 | -0.07  (-0.36, 0.23) | 0.66 |
| Breaks in SB, times/day | -0.15  (-0.49, 0.18) | 0.37 | -0.01  (-0.32, 0.30) | 0.94 |
| Standing,  h/day | -0.07  (-0.41, 0.27) | 0.70 | 0.10  (-0.30, 0.23) | 0.51 |
| Standing,  % of daily wear time | -0.06  (-0.39, 0.28) | 0.74 | 0.08  (-0.22, 0.38) | 0.60 |
| Steps, number/day | -0.31  (-0.61, -0.01) | **0.045** | -0.1  (-0.41, 0.22) | 0.53 |
| LPA,  h/day | 0.02  (-0.30, 0.34) | 0.89 | 0.09  (-0.19, 0.38) | 0.51 |
| LPA,  % of daily wear time | 0.04  (-0.28, 0.35) | 0.82 | 0.08  (-0.20, 0.36) | 0.57 |
| MVPA,  h/day | -0.24  (-0.55, 0.06) | 0.12 | -0.05  (-0.36, 0.26) | 0.74 |
| MVPA,  % of daily wear time | -0.23  (-0.54, 0.07) | 0.13 | -0.06  (-0.36, 0.24) | 0.68 |
| PA,  h/day | -0.11  (-0.42, 0.19) | 0.45 | 0.05  (-0.24, 0.33) | 0.74 |
| PA,  % of daily wear time | -0.11  (-0.41, 0.20) | 0.48 | 0.03  (-0.25, 0.31) | 0.84 |
| **Abbreviations:** LFC = liver fat content, LPA = light physical activity, MUFA = monounsaturated fatty acids, MRI = magnetic resonance imaging, MVPA = moderate to vigorous physical activity, PA = physical activity (LPA and MVPA together), SB =sedentary behaviour (sitting and lying).  **Model 1 adjusted for age and sex.**  **Model 2 adjusted for age, sex and body fat %.** | | | | |

| **Table 2. Age-, sex- and body fat % -adjusted linear mixed regression estimates (standardized β coefficients (95% CI)) between MRI-measured LFC, fitness and dietary intake.** | | | | |
| --- | --- | --- | --- | --- |
|  | **LFC MRI (%)** | | | |
|  | **Model 1** | | **Model 2** | |
|  | **β** | **p** | **β** | **p** |
| VO_2max_,  ml/min/kg | -0.50  (-0.84, -0.14) | **0.007** | -0.15  (-0.60, 0.30) | 0.51 |
| VO_2max_, ml/min/kg_FFM_ | -0.08  (-0.45, 0.28) | 0.65 | -0.06  (-0.38, 0.26) | 0.71 |
| Maximal load, W | -0.21  (-0.59, 0.18) | 0.29 | 0.03  (-0.34, 0.39) | 0.89 |
| Total EI, kcal/day | 0.17  (-0.14, 0.49) | 0.27 | 0.16  (-0.11, 0.44) | 0.24 |
| Protein, % of daily EI | -0.23  (-0.53, 0.06) | 0.12 | -0.30  (-0.55, -0.05) | **0.02** |
| Carbohydrates, % of daily EI | -0.11  (-0.42, 0.2) | 0.48 | 0.03  (-0.25, 0.32) | 0.81 |
| Fat,  % of daily EI | 0.21  (-0.1, 0.52) | 0.18 | 0.09  (-0.20, 0.37) | 0.54 |
| Alcohol,  % of daily EI | 0.11  (-0.2, 0.41) | 0.48 | 0.12  (-0.14, 0.39) | 0.35 |
| SFA,  % of daily EI | -0.13  (-0.45, 0.19) | 0.42 | -0.05  (-0.33, 0.24) | 0.73 |
| MUFA,  % of daily EI | 0.30  (0.01, 0.59) | **0.046** | 0.14  (-0.14, 0.43) | 0.32 |
| PUFA,  % of daily EI | 0.25  (-0.04, 0.54) | 0.09 | 0.10  (-0.18, 0.38) | 0.46 |
| Saccharose,  % of daily EI | 0.09  (-0.21, 0.39) | 0.54 | 0.16  (-0.10, 0.43) | 0.22 |
| **Abbreviations:** EI = energy intake, FFM = fat free mass, LFC = liver fat content, MUFA = monounsaturated fatty acids, MRI = magnetic resonance imaging, PUFA = polyunsaturated fatty acids, SFA = saturated fatty acids, VO_2max_ = maximal oxygen consumption.  **Model 1 adjusted for age and sex.**  **Model 2 adjusted for age, sex and body fat %.** | | | | |

| **Table 3. Age-, sex- and body fat %-adjusted linear mixed regression estimates (standardized β coefficients (95% CI)) between MRI-measured LFC, body composition and cardiometabolic risk factors.** | | | | |
| --- | --- | --- | --- | --- |
|  | **LFC MRI (%)** | | | |
|  | **Model 1** | | **Model 2** | |
|  | **β** | **p** | **β** | **p** |
| Body fat, % | 0.62  (0.27, 0.97) | **0.001** |  |  |
| Waist, cm | 0.43  (0.15, 0.71) | **0.004** | 0.20  (-0.17, 0.56) | 0.28 |
| BMI, kg/m2 | 0.26  (-0.04, 0.55) | 0.085 | -0.14  (-0.52, 0.24) | 0.46 |
| SBP, mmHg | -0.09  (-0.41, 0.23) | 0.57 | -0.19  (-0.46, 0.09) | 0.18 |
| DBP, mmHg | 0.18  (-0.12, 0.48) | 0.24 | 0.08  (-0.2, 0.35) | 0.56 |
| Resting heart rate, bpm | -0.04  (-0.26, 0.35) | 0.77 | 0.04  (-0.21, 0.29) | 0.75 |
| BP medication | -0.03  (-0.36, 0.3) | 0.85 | -0.03  (-0.32, 0.25) | 0.82 |
| Cholesterol medication | -0.03  (-0.33, 0.27) | 0.84 | 0.07  (-0.21, 0.34) | 0.63 |
| f-Glucose, mmol/l | -0.11  (-0.2, 0.42) | 0.49 | 0.05  (-0.22, 0.33) | 0.71 |
| f-Insulin, mU/l | 0.39  (0.09, 0.68) | **0.01** | 0.22  (-0.08, 0.52) | 0.15 |
| HOMA-IR | 0.38  (0.08, 0.67) | **0.02** | 0.21  (-0.09, 0.51) | 0.17 |
| M-value, mg/kg/min | -0.39  (-0.66, -0.13) | **0.005** | -0.19  (-0.52, 0.14) | 0.2487 |
| HbA1c, mmol/mol | 0.55  (0.28, 0.81) | **0.0001** | 0.46  (0.21, 0.70) | **0.0006** |
| Triglycerides, mmol/l | 0.32  (0.04, 0.6) | **0.03** | 0.3  (0.06, 0.55) | **0.02** |
| Cholesterol, mmol/l | 0.18  (-0.12, 0.49) | 0.23 | 0.20  (-0.06, 0.47) | 0.13 |
| HDL, mmol/l | -0.14  (-0.47, 0.19) | 0.40 | -0.09  (-0.38, 0.21) | 0.55 |
| LDL, mmol/l | 0.16  (-0.14, 0.46) | 0.28 | 0.16  (-0.1, 0.43) | 0.22 |
| ALT, U/l | 0.51  (0.25, 0.78) | **0.0003** | 0.41  (0.15, 0.66) | **0.002** |
| AST, U/l | 0.39  (0.07, 0.71) | **0.02** | 0.33  (0.05, 0.61) | **0.02** |
| GGT, U/l | 0.29  (-0.01, 0.58) | 0.055 | 0.23  (-0.03, 0.49) | 0.086 |
| **Abbreviations;** ALT = alanine aminotransferase, AST = aspartate aminotransferase, BP =blood pressure, DBP =diastolic blood pressure, GGT = γ-glutamyltransferase, HbA_1c_ = hemoglobin A_1c_, HOMA-IR = homeostatic model assessment for insulin resistance, LFC = liver fat content, MRI = magnetic resonance imaging, M-value = whole-body insulin sensitivity, SBP = systolic blood pressure.  **Model 1 adjusted for age and sex.**  **Model 2 adjusted for age, sex and body fat %.** | | | | |
